# Antagonistic effects of predator color morph abundance and saliency on prey anti-predator responses

**DOI:** 10.1093/beheco/araf059

**Published:** 2025-05-24

**Authors:** S R Matchette, J Schneider, C Drerup, S Winters, A N Radford, J E Herbert-Read

**Affiliations:** Department of Zoology, University of Cambridge, Downing Street, Cambridge, CB2 3EJ, United Kingdom; Reef Renewal Foundation Curaçao, Ocean Encounters, Babor Kibra z/n, Willemstad, Curaçao; Department of Freshwater and Marine Ecology, University of Amsterdam, Sciencepark 904, 1098 XH, Amsterdam, Netherlands; Department of Zoology, University of Cambridge, Downing Street, Cambridge, CB2 3EJ, United Kingdom; Department of Biosciences, Durham University, South Road, Durham, DH1 3LE, United Kingdom; Organismal and Evolutionary Biology Research Program, Faculty of Biological and Environmental Sciences, University of Helsinki, Viikinkaari 1, 00790, Helsinki, Finland; School of Biological Sciences, University of Bristol, 24 Tyndall Avenue, Bristol, BS8 1TQ, United Kingdom; Department of Zoology, University of Cambridge, Downing Street, Cambridge, CB2 3EJ, United Kingdom

**Keywords:** abundance, colouration, polymorphism, search image, saliency

## Abstract

The color polymorphisms of prey species are often maintained by apostatic selection. In particular, rarer morphs are thought to be at an advantage because attentional constraints result in predators forming search images, which are based on the most abundant prey morph. Predatory species can also be polymorphic and predator morph abundance may be maintained by a similar mechanism, given prey are also likely to form search images to ensure fast and appropriate anti-predatory responses. Alternatively, given that the predator polymorphism may be driven by other ecological factors (eg niche divergence or sexual selection), prey may instead be highly sensitive to the relative visual saliency of different predatory morphs, which in turn could impact predator morph abundance. Here, by combining empirical observations with a field experiment, we assessed how the relative abundance and saliency of different color morphs of the predatory trumpetfish (*Aulostomus maculatus*) influenced the behavioral responses of a typical prey species, the bicolor damselfish (*Stegastes partitus*). We found that more abundant predator color morphs were less salient in damselfish vision (relative to the background) than less abundant color morphs. By presenting 3D models of each morph to damselfish, we found that they did not respond differently to more abundant or more salient morphs. Our results suggest that both the relative abundance and saliency of predator morphs could contribute towards the search images used by prey. Specifically, each morph could have relatively equal detectability if their abundance and saliency have antagonistic effects on search-image formation in prey.

## Teaser

Predators can have different color morphs, but whether morph abundance or saliency is more influential in shaping antipredator behavior in prey remains unclear. By studying the behavioral responses of damselfish to 3D models of different predatory trumpetfish color morphs, we find that morph abundance and saliency may balance each other out in search-image formation.

## Introduction

Colour polymorphisms, whereby multiple discrete genetically determined phenotypes coexist in a single interbreeding population (White and Kemp 2016), are a common phenomenon across the animal kingdom (Bond 2007). For example, the wings of the box tree moth (*Cydalima perspectalis*) can be brown or pearly white (Poloni et al. 2024), aposematic strawberry poison-dart frogs (*Dendrobates pumilio*) adopt bright red to dull green colourations (Wang and Shaffer 2008), and male side-blotched lizards *(Uta stansburiana*) can have one of three different throat markings (Sinervo and Lively 1996). While natural selection is expected to remove less successful morphs from populations (Karpestam et al. 2016), the maintenance of color morphs can be driven by several mechanisms (Robinson et al. 2024), including assortative mating (Healey et al. 2008) and niche divergence (Stevens et al. 2014).

Our understanding of color polymorphisms arises primarily from studies on prey species (Lerner et al. 1956; Endler 1988; Bond and Kamil 2002; Karpestam et al. 2013, 2016; Troscianko et al. 2018), yet many polymorphic species are predatory (Paulson 1973; Passarotto et al. 2018; Rodríguez-Morales et al. 2018; Ajuria Ibarra et al. 2019). For prey species, color polymorphisms can be maintained via apostatic selection, whereby the survival of a morph is related to its abundance relative to other morphs in the population (Lerner et al. 1956; Endler 1988; Bond and Kamil 2002; Bond 2007; Karpestam et al. 2016; White and Kemp 2016; Troscianko et al. 2018, 2021). In particular, less common forms are thought to be at a selective advantage over more common forms, because of the attentional constraints of predators (van Leeuwen and Jansen 2010) that use the visual appearance of prey in recent encounters as a “search image” to aid the detection of their next prey (Pietrewicz and Kamil 1979; Rohwer 1983; Rohwer and Paulson 1987; Dukas and Kamil 2001). Given that search images are based on the most frequently encountered morphs (Rohwer 1983; Bond and Kamil 2002), rarer morphs may be less likely to be detected or recognized, thereby surviving and reproducing until they themselves become more common (White and Kemp 2016). In principle, predator polymorphisms may be maintained by similar mechanisms to prey (Rohwer and Paulson 1987; Robinson et al. 2024). Indeed, a predator’s foraging success often hinges on its ability to go undetected or unrecognized until it is close enough to strike (Cooper and Frederick 2007; Pembury Smith and Ruxton 2020). If the maintenance of a predator’s polymorphism in a population is driven by interactions with prey—with prey forming search images of their predators to ensure fast and appropriate anti-predatory responses—then apostatic selection could act (Rohwer and Paulson 1987). If so, the relative abundances of different predatory phenotypes within a population may influence the type and magnitude of a prey’s response to each predator morph, with prey being less likely to detect and respond to rarer compared to more common predator morphs.

If search images and apostatic selection determine the abundance of different morphs in populations, then all morphs are expected to have relatively low saliency or detectability against their backgrounds (Bond and Kamil 2002; Bond 2007; Poloni et al. 2024). This is because no matter their rarity, conspicuous morphs are likely to be detected, and therefore selection should favor all morphs to be inconspicuous but discrete from each other. Bond and Kamil (2002) experimentally demonstrated this using foraging jays (*Cyanocitta cristata*) and digitally evolving moth prey, highlighting that predation selects to increase the degree of polymorphism within cryptic but not conspicuous prey populations. In some polymorphic predator populations, however, it appears that some morphs are relatively more salient than others. For instance, the gold morph of the Neotropical cichlid fish, *Amphilophus labiatus*, is more conspicuous than its dark counterpart (Sowersby et al. 2015; Lehtonen et al. 2023), which will influence its detectability to both its predators (Torres-Dowdall et al. 2014) and the fish it preys upon. This difference in saliency may be expected because some color polymorphisms in predators are likely to arise not just as a result of apostatic selection but because of other processes, such as mate choice, thermoregulation or niche divergence. In general, we might expect predator morphs that are relatively more conspicuous to be more easily detected by prey. And, unlike conspicuous prey morphs that are likely to be extirpated, more conspicuous predatory morphs may not necessarily be completely removed from populations due to the life–dinner principle (Dawkins and Krebs 1979). That is, prey are under greater selective pressure than predators because, if detectable, conspicuous prey may lose their life whereas conspicuous predators may only lose a meal. Nevertheless, we might expect more salient predatory morphs to be relatively less abundant in populations if the saliency of a predator reduces its success in attacks.

**Fig. 1.**
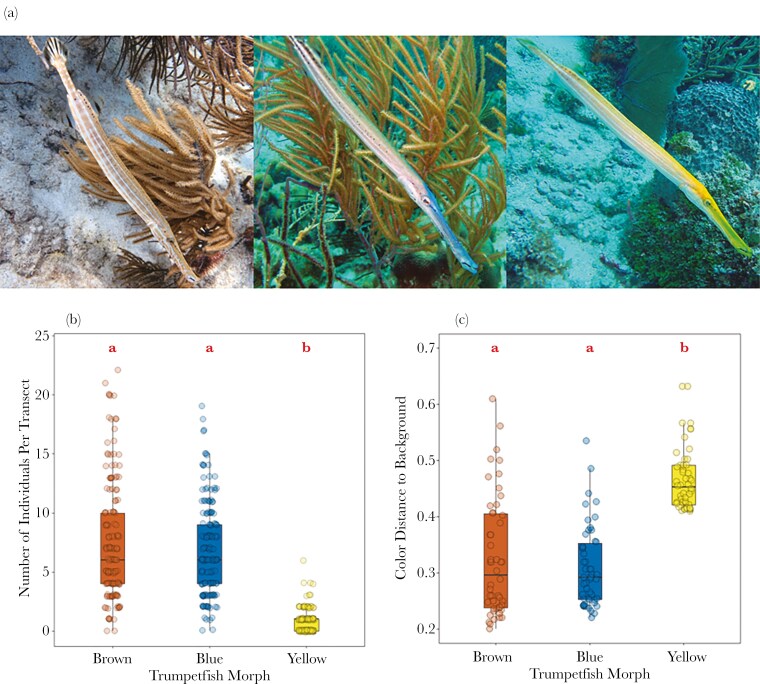
(A) Photographs of the three color morphs of trumpetfish (*Aulostomus maculatus*): individuals occur with a reddish-brown body and head (left; photograph by James St. John licensed under CC BY 2.0, cropped by the authors), a silvery-gray body and blue head (middle; photograph by Pauline Walsh Jacobson licenced under CC BY 4.0, cropped by the authors), or a xanthic (yellow) body and head (right; photograph by Pauline Walsh Jacobson licenced under CC BY 4.0, cropped by the authors). (B) The number of each trumpetfish color morph observed on a given transect (n = 158 transects). (C) The saliency of each morph when the color of each model (as viewed in anemonefish color space, our prey species proxy) was compared to a set of 50 different blue backgrounds using the *getColorDistanceMatrix* function in the *colordistance* package (Weller and Westneat 2019). The lower the color distance, the higher the similarity between the model colouration and a given blue background. (B-C) The box plots show the median and 25th and 75th percentiles; the whiskers indicate the values within 1.5 times the interquartile range. The hollow circles represent the raw data points. Red letter labels above the boxes denote the pairwise comparisons between color morphs, whereby morphs with the same letter do not statistically differ.

By combining natural observations with a field experiment, we aimed to investigate how the relative abundance and saliency of different color morphs of the predatory trumpetfish (*Aulostomus maculatus*) influence the responses of a typical prey species, the bicolor damselfish (*Stegastes partitus*). Trumpetfish are common predators on coral reefs across the Caribbean, preying on a diversity of small fish (Randall 1967) using several different foraging strategies (Randall 1968; Kaufman 1976; Aronson 1983; DeLoach et al. 2019). Trumpetfish occur in one of three color morphs, which do not appear to be plastic (*personal observation*), nor related to sex (Lochmann 1989) or body size (*personal observation*): individuals can have a reddish-brown body and head (hereafter, “brown”), a silvery-gray body with a blue head (hereafter, “blue”), or a xanthic body and head (hereafter, “yellow”; Fig. 1A) (DeLoach et al. 2019). We first used detailed observations to establish the relative abundance of the three trumpetfish color morphs across several field sites. We then created realistic 3D models of each trumpetfish color morph and assessed the saliency of each trumpetfish color morph, before presenting these models to natural colonies of bicolor damselfish to quantify any differences in anti-predator responses. Bicolor damselfish exhibit strong and characteristic behavioral responses to predators, including trumpetfish (Helfman and Winkelman 1997; Matchette et al. 2023), and are predicted to have good color vision (Stieb et al. 2016, 2017; Cortesi et al. 2020). We predicted that if prey use search images based solely on morph abundance, then rarer morphs should be less likely to be detected than more common morphs. On the other hand, if the saliency of morphs affects their likelihood of being detected, then damselfish should be more likely to detect and respond to more salient morphs. In this case, we might also expect an inverse relationship between morph saliency and morph abundance.

## Methods

### Trumpetfish abundance

To assess the overall abundance of each trumpetfish morph, we conducted a total of 158 linear transects across nine locations (minimum of 10 transects per location; see Supplementary Table S1) on reefs surrounding Curaçao, Netherland Antilles. Transects were conducted between April 2022 and December 2022 (n = 113), and between March 2023 and May 2023 (n = 45). Transects comprised a researcher on SCUBA swimming, continuously and at a steady rate, parallel with the reef crest for 15 min, roughly 5 m above the reef floor, recording via camera every individual trumpetfish within ~15 m either side of the transect bearing. At each location, the transect started and ended at two predetermined landmarks, with the overall distance translating to ~200 m stretches of the reef. Over the course of the study, two researchers occupied the surveyor role when conducting transects; to ensure consistency between researchers (eg in swimming speeds and ability to detect trumpetfish), they conducted a set of pilot transects together prior to the start of the study. The researcher filmed the transect continuously using two GoPro cameras (Hero 10; 3840 × 2160 px, 30 fps, linear angle) fixed to a stereo camera rig (Neewer; Shenzhen, Guangdong, China), held out at a 45-degree angle. For every trumpetfish encountered, the researcher pointed the cameras towards the individual for ~2 s. The assessment of the color of each trumpetfish encountered was accomplished in situ, relayed to the recording using a hand signal that was unique to each morph. While the swimming speed and directed trajectory of the researcher minimized the chance of recording the same trumpetfish twice, a second researcher helped track trumpetfish that had recently been counted and informed the lead researcher if they were likely to appear back in shot. All encounters (and the corresponding color) were collated from the videos post-hoc in the software Loopy (Loopbio, Vienna, Austria; www.loopbio.com/loopy/).

## Behavioural experiment

### 3D model generation

We used a digital 3D model of trumpetfish, identical to the one used by Matchette et al. (2023) and previously acquired from TurboSquid (product ID: 1251130; TurboSquid, New Orleans, LA, USA), to create physical 3D models of trumpetfish. This digital model was manipulated in Blender (Blender v.3.1.2, Amsterdam, Netherlands), FreeCAD (FreeCAD v.0.19) and PrusaSlicer (v2.3.3; Prusa Research, Prague, Czech Republic), and was 3D printed using an Original Prusa i3 MK3S + (Prusa Research, Prague, Czech Republic) with clear 1.75 mm PETG filament (TecBears, Kwun Tong, Hong Kong). The final 3D model (hereafter, ‘model’) had a total length of 55.5 cm, which falls within the natural size range of *Aulostomus maculatus* (Humann and DeLoach 2008). To reduce pseudo-replication, we printed three models for each of the three color morph treatments (nine models in total).

### 3D model coloration

To apply the correct coloration to each model, we created a 2D digital representation of the trumpetfish skin that could be printed on waterproof paper (ToughPrint) and wrapped around the physical 3D model. This technique was made possible by using the software Pepakura Designer (Tamasoft; Tokyo, Japan) and has previously been implemented in behavioral research to generate realistic animal models (Mesken et al. 2022). To best replicate the real colors of each morph in the digital skins, we analyzed standardized images of three fresh trumpetfish cadavers (one of each morph). This analysis reflected a typical image-processing pipeline (Stevens et al. 2007; Troscianko and Stevens 2015) and used a bespoke MATLAB (The Mathworks Inc; Natick, MA, U.S.A.) script, which can be found in the open-source *TrumpetfishColour* repository (Winters 2025) . In summary, the cadaver images were transformed in to the color space of an ecologically relevant visual system, with the closest available proxy to the bicolor damselfish being the anemonefish *Amphiprion akindynos*, an Indo-Pacific species that is also a member of the Pomacentridae family (Siebeck and Marshall 2007; Hofmann et al. 2012; Stieb et al. 2019; Cortesi et al. 2020). We then extracted the mean color from five regions of the transformed trumpetfish image, which were used as the color palette when coloring a manually-created digital skin of each morph in GIMP (v.2.10.30). To account for any major change in color when printed, the colors of each printed digital skin were compared to their pre-printed values. Once these comparisons were within one JND of each other (Vorobyev and Osorio 1998; Vorobyev et al. 2001; Siddiqi et al. 2004), each digital skin was printed, wrapped around the models and fixed in place with super glue (Supplementary Figure 1). To reduce any possible paper (and color) degradation, we applied two successive coats of nontoxic waterproof epoxy resin to each wrapped model. Specific details for the image transformation, color extraction and color validation can be found in the Supplementary Materials.

### 3D model color saliency

To assess how detectable each color morph is likely to be to their prey (damselfish), we calculated the saliency of each 3D printed model relative to the background midwater. In this context, as inferred by Marshall et al. (2003), the yellow trumpetfish morphs were predicted to be more salient than the brown or blue trumpetfish morphs. By using our 3D printed models, instead of trumpetfish in situ (which would require a specialized waterproof color standard) or the colors extracted from the trumpetfish cadavers (as outlined above), we were able to relate our saliency measurements directly to the stimuli presented to damselfish in the experiment. In summary, we used the *getHistList* function in the *colordistance* package (Weller and Westneat 2019) to extract a condensed color palette from the transformed image of each morph model, which were then compared to a set of blue hue images (n = 50; Supplementary Figure S2). The latter were chosen by eye and were deemed to act as the likely background when each trumpetfish model was presented to damselfish in the experimental set-up (see below). Using the *getColorDistanceMatrix* function with the “earth mover’s distance” method (Weller and Westneat 2019), we then generated a color distance matrix by comparing each binned morph model image with each blue hue image in turn. For each morph–blue image comparison (n = 50 comparisons per morph), this function essentially compares the distribution of color histograms between the two images and generates an overall color similarity score (“color distance” - the lower the score, the more similar the colors are), which was stored for later analysis. This method was preferred for analyzing model saliency as it takes in to account the relative proportion of colors present in each model, which will be a significant component of each model’s overall saliency. Further information regarding the color saliency analysis can be found in the Supplementary Materials.

It must be noted that the making of the models and the saliency analysis did not account for UV sensitivity, despite many damselfish species being sensitive to UV light (McFarland and Loew 1994; Cortesi et al. 2020), which they can use for intraspecific communication (Siebeck et al. 2010) and/or zooplankton detection (Stieb et al. 2017). While our models did not fluoresce or brighten when subjected to a handheld LED UV flashlight (395 nm, 100 LED), a common technique to assess UV reflectance (see Sobral and Souza-Gudinho 2022), the role of UV sensitivity in the trumpetfish–damselfish system remains unknown and of interest for future study.

### Experimental set-up and procedure

**Fig. 2.**
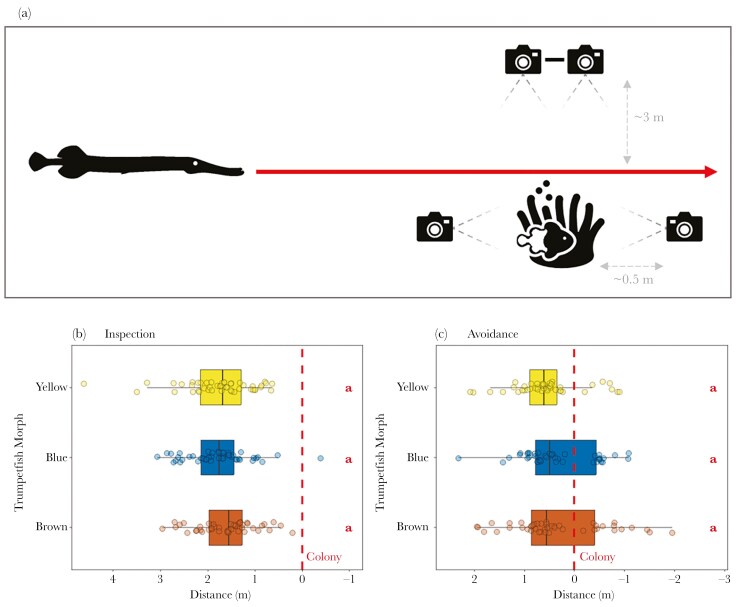
(A) Schematic detailing the experimental set-up. For each presentation, a researcher attached a trumpetfish model to the reeling line, at which point the other researcher hand-reeled the model along the line past the damselfish colony (direction of travel denoted by the red line). We positioned two cameras next to the colony to record the behavioral responses of the damselfish, while we fixed two further cameras to a stereo camera rig used to extract the distance between the model and the colony post-hoc. The distance between each model and the colony (represented by the red dotted line; n = 48 colonies) at the time when the first inspection behavior (B) and the first avoidance behavior (C) was exhibited. (B-C) The direction of travel of the model is from left to right such that positive values indicate the model approaching the colony, while negative values indicate the departure of the model from the colony. Box plots show the median and 25th and 75th percentiles; the whiskers indicate the values within 1.5 times the interquartile range. The hollow circles represent the raw data points. Red letter labels to the right of each plot denote the pairwise comparisons between color morphs, whereby morphs with the same letter do not statistically differ.

Bicolor damselfish live in groups among semi-isolated structures on the reef floor (hereafter, ‘colony’) (Helfman and Winkelman 1997). Overall, while on SCUBA, we presented 48 bicolor damselfish colonies with each of the three color morph treatments in a balanced randomized block design and filmed their behavioral responses (Fig. 2A). Presentations involved hand-reeling each model between two tripods, passing over the colony halfway between the two (as in Matchette et al. 2023). Post-hoc analysis was conducted to ensure that the speed (and the variation in the speed) of hand-reeling was consistent across treatments (see Supplementary Materials). While trumpetfish may spend some time hovering vertically near to colonies (defined as their “strike” posture), trumpetfish largely adopt a “search” posture, swimming horizontally across the reef (Helfman 1989; Helfman and Winkelman 1997); we therefore presented models in this way to the damselfish to best represent a foraging trumpetfish. We positioned two cameras (“Focal”; GoPro Hero 10; 3840 × 2160 px, 30 fps, linear angle) on either side of the colony to capture the damselfish behavior; one captured the approach of the model and the other captured its departure. We fixed two further cameras (“Stereo”; GoPro Hero 10; 3840 × 2160 px, 30 fps, linear angle) to a stereo camera rig (left and right) that was positioned ~3 m away, perpendicular to the nylon line, to capture the full transition of the model from tripod to tripod. All cameras were synchronized using a series of clear “tank bangs”: a ball on an elasticated band which dinged against the SCUBA air tank. We gave each colony 5 mins to acclimate to the presence of the nearby equipment before the start of each presentation. We conducted the experiment on 12 colonies in each of four locations on reefs surrounding Curaçao, Netherland Antilles (locations 1 to 4 in Supplementary Table S1). All three color morphs have been observed hunting bicolor damselfish across all four locations (*personal observations*). Presentations were conducted between 9:00 am and 11:00 am by the same two researchers on SCUBA. All procedures were approved by the University of Cambridge Animal Welfare and Ethical Review Body (0S2022/13).

### Video processing

We first synchronized the videos from both the focal and stereo cameras and then trimmed them to include only segments containing the model presentations; the tank bang at the start of each presentation was located and used to isolate the subsequent presentation for each of the four cameras for all presentations. We then scored damselfish abundance (the maximum number of fish observed on the colony) and behavior present in the focal presentation videos in Loopy.

As a proxy for the detectability of each model, we used inspection behavior given that this behavior typically indicates the first awareness of the approaching model, with more inspections indicating increased detectability. Inspection behavior by a colony was defined as at least one individual facing with and swimming towards the model, as observed in guppies (Godin and Davis 1995) and damselfish (Matchette et al. 2023). Using the stereo camera videos, as in Matchette et al. (2023), we extracted the position of the model trumpetfish relative to the colony (ie the distance between the model and the nearest point of the colony structure, and whether the model was approaching or departing) at the time when the first inspection behavior was exhibited (“inspection distance”). We also calculated the maximum number of fish in the colony exhibiting inspection behavior at any given time.

To test if different morphs were perceived to be more or less threatening, we also recorded the avoidance responses of damselfish. Avoidance responses involved at least one individual fleeing rapidly towards the colony shelter. Bicolor damselfish are highly reactive to avoidance responses of conspecifics (*personal observation*); ie the initial response from one individual typically sparks the rapid movement of other conspecifics, usually within ~100 milliseconds (2 to 3 video frames). As a result, where colonies exhibit multiple avoidances, we defined independent avoidance responses as those occurring more than 0.5 s (15 video frames) from another (as in Matchette et al. 2023). In particular, we recorded the distance at which the first avoidance response by damselfish occurred and the number of avoidance responses exhibited by fish.

### Statistical analysis

All statistical analyses were performed in R (v. 3.3.2), and we used independent linear mixed models (LMMs) and generalized linear mixed models (GLMMs) to analyze the influence of trumpetfish color morph upon each component of the investigation (summarized in Supplementary Table S2). A GLMM was used to compare the observation frequency of each trumpetfish color morph (Poisson family), with transect location included as a random effect, denoted in *lme4* syntax as (1 | location). An LMM was used to compare the color distance scores generated between each color morph and a set of blue backgrounds, with the ID of each blue hue included as a random effect, denoted in *lme4* syntax as (1 | hue ID).

For models analyzing the behavioral responses of damselfishes in the behavioral experiment, the fixed effects included color morph (categorical) and treatment order (ordinal). Independent LMMs were used to assess how our fixed effects influenced the distance when each trumpetfish model was first inspected and the distance when each trumpetfish was first avoided (square-root transformation). Independent GLMMs were used to assess how our fixed effects influenced the maximum proportion of the colony that inspected each trumpetfish model (binomial) and the number of avoidance responses exhibited (Poisson). For all behavioral response models, colony ID was provided as a nested random effect within location, denoted in *lme4* syntax as (1 | location / colony ID). To check the assumptions of each statistical model, the *DHARMa* package (Hartig and Lohse 2022) was used to interpret the dispersion and distribution of the residuals. In all models, when color morph was found to be significant, pairwise comparisons were computed using the *emmeans* function (with “Tukey” contrasts) from the *emmeans* package (Lenth et al. 2020).

## Results

The relative abundance of each morph in the population differed (GLMM: LRT = 1165.30, df = 2, *P* < 0.001; Supplementary Table S2; Fig. 1B). Yellow morphs were rarer than brown morphs (Tukey: z.ratio = 24.03, *P* < 0.001) and blue morphs (z.ratio = 22.93, *P* < 0.001), with blue and brown morphs having similar abundances (z.ratio = 2.26, *P* = 0.062).

The color of the trumpetfish morphs used in model presentations differed in their saliency relative to a set of blue backgrounds (LMM: LRT = 92.07, df = 2, *P* < 0.001; Supplementary Table S2; Fig. 1C). The yellow models were more dissimilar to the background than both the brown models (Tukey: t.ratio = -9.89, *P* < 0.001) and the blue models (t.ratio = -11.01, *P* < 0.001), while the blue and brown models did not differ significantly in their relative saliency (t.ratio = 1.12, *P* = 0.505).

There was no evidence that any of the morphs were more likely to be detected than the others. The distance between the model and the colony when the first inspection behavior occurred was not significantly different between morphs (LMM: LRT = 2.17, df = 2, P = 0.338; Supplementary Table S2; Fig. 2B), nor was the maximum proportion of a colony that inspected the morphs (GLMM: LRT = 5.37, df = 2, *P *= 0.068; Supplementary Figure S3A). There was also no evidence that damselfish treated each morph as a different threat level, as the distance at which the damselfish first exhibited an avoidance response was not influenced by the color of the trumpetfish models (LMM: LRT = 2.17, df = 2, *P *= 0.337; Fig. 2C). Similarly, the number of avoidance responses by damselfish did not differ significantly between the models (GLMM: LRT = 5.32, df = 2, P = 0.070; Supplementary Figure S3B). Overall, therefore, there was no evidence that more common morphs (blue or brown), or more salient morphs (yellow), were more detectable or perceived to be more threatening than others.

## Discussion

We tested whether the abundance or saliency of different predatory trumpetfish color morphs affected their likelihood of being detected and evaded by bicolor damselfish. While yellow trumpetfish morphs were rarer than the blue or brown morphs, the yellow morph was also likely more salient than the other two morphs to damselfish prey. Contrary to our predictions, the behavioral responses of damselfish did not differ to the presentation of different trumpetfish color morphs in situ. Specifically, no single morph was detected or responded to earlier or later than the others, with damselfish also exhibiting similar avoidance behaviors to all morphs. These findings could be explained by differences in the mechanisms behind search-image formation in prey compared to predators, as well as the other possible drivers underlying the evolution and maintenance of trumpetfish color polymorphism.

We expected more abundant or more salient morphs to be more easily detectable, but instead found that damselfish detected rarer, more salient morphs (yellow morphs) as readily as more common, less salient morphs (blue and brown morphs). When cryptic prey are polymorphic, all morphs are expected to have low saliency, as conspicuous prey are easier to detect regardless of how different they are to others (Bond and Kamil 2002). Search-image formation in predators is therefore driven primarily by the frequency at which different cryptic prey morphs are encountered (Rohwer 1983; Bond and Kamil 2002). The same mechanism may not be true for how prey species form search images. In predators, different morphs may not necessarily have the same levels of crypticity, as polymorphisms may exist for reasons other than apostatic selection. Indeed, sexual signaling and mate choice are key drivers in the maintenance of bright color polymorphisms in many prey species (eg Elmer et al. 2009; Huang et al. 2014; Estévez et al. 2020; Lehtonen et al. 2023). Prey searching for polymorphic predators with different conspicuousness may, therefore, form search images that not only rely on the abundance of different predator morphs encountered, but also their saliency. This could result in relatively lower frequencies of conspicuous morphs and relatively higher frequencies of cryptic morphs if both saliency and frequency (via search–image formation) contributed to detectability. At some equilibrium point, we might expect the relatively frequencies of morphs with different saliencies to have the same detectability, if search images were focused on detecting more common forms. The equilibrium between saliency and abundance provides a potential explanation for our results that there was no difference in damselfish responses to each of the different color morphs.

While our understanding of predatory polymorphisms arises largely from avian species, particularly birds of prey (Rohwer and Paulson 1987; Fowlie and Krüger 2003; Passarotto et al. 2018; Robinson et al. 2024), the underlying mechanism for the emergence and maintenance of polymorphisms in predatory species may not involve interactions with their prey. For example, the relative rarity of yellow trumpetfish morphs may simply be driven by genetic infrastructure, such as allele homozygosity (Nokelainen et al. 2022), and different morphs may persist due to assortative or disassortative mating, neutral processes, historical bottlenecks, secondary contact, physiological demands (Clotfelter et al. 2007) or an interaction between multiple mechanisms (Robinson et al. 2024). Indeed, while less common, differences in trumpetfish appearance and abundance may also be driven by interactions with their own predators, which may include groupers and snappers (Randall 1967), before reaching a size that exceeds the gape limitations of these predators. Alternatively, color polymorphism in trumpetfish may be driven by interactions with prey, but through mechanisms other than search-image formation, such as disruptive selection. Here, each morph could instead occupy a different predatory niche, hunting different prey species or hunting at different times of the day, as seen in owls (Passarotto et al. 2018) and crab spiders (Rodríguez-Morales et al. 2018). For example, despite observations of all trumpetfish morphs hunting bicolor damselfish, different morphs may typically target different prey species. Indeed, though statistically non-significant, there was a trend towards yellows inducing a greater proportion of the colony to inspect and a greater number of avoidance responses, which may indicate that these morphs are more threatening to bicolor damselfish in this context. Furthermore, the saliency of an animal against a background is entirely defined by the vision of the viewer (Bond 2007), and thus the appearance of each morph will likely differ between prey species. The responses of different species other than bicolor damselfish to different trumpetfish color morphs remains to be investigated.

The foraging strategy of morphs may also differ. In particular, rather than approaching prey from across an open reef (as in the current experiment), some morphs may use structures like gorgonians or complex hard corals to approach their prey stealthily, or adopt a sit-and-wait strategy. Indeed, while the yellow coloration in reef fish is predicted to be highly salient when viewed against midwater blues, this color can actually appear well-camouflaged when viewed against the average color of the reef (Marshall 2000), as illustrated in the yellow phase of the Chinese trumpetfish (*Aulostomus chinensis*) (Marshall et al. 2003). With the availability of many heterospecifics on a coral reef, some morphs may also be more likely to exhibit shadowing behavior, whereby an individual swims alongside other fish when foraging (Kaufman 1976; Aronson 1983; Matchette et al. 2023). Further investigation is required to test the mechanistic and functional explanations of the color polymorphism of the predatory trumpetfish, including a greater understanding of how each morph differs in their wider ecology and how colouration is influenced at a genomic level.

Another reason why damselfish may not have responded to each of the three color morphs differently is that damselfish may not use color cues to detect an approaching predator. Indeed, prey may rely on non-chromatic cues when detecting threats, such as size (Hemmi 2005; Cooper and Stankowich 2010) or movement (Llandres and Rodríguez-Gironés 2011). Trumpetfish have a characteristic long silhouette, and bicolor damselfish are also “hypersensitive” to trumpetfish that are positioned as if they are foraging (ie parallel to the water surface) (Helfman and Winkelman 1997). Such an effect may be accentuated by our experimental set-up with models being presented to colonies in midwater, where their silhouettes are likely to be more distinctive. If the silhouette and/or posture of trumpetfish are particularly reliable cues for other animals, then this may explain why trumpetfish exhibit behaviors that typically occlude or disrupt these features. For example, trumpetfish are frequently observed hovering vertically in the water-column, which reduces the number of approaches by damselfish (Helfman 1989), or sheltering amongst soft corals (Randall 1968; Aronson 1983). Shadowing behavior, too, allows individuals to get closer to their prey without being detected (Matchette et al. 2023), presumably because it occludes or disrupts the trumpetfish silhouette. Prey reliance on non-chromatic cues may also relax the selection on trumpetfish colouration for concealment, allowing this phenotype to evolve for other functions, such as in sexual selection (Endler 1983; Hodge et al. 2020) or for physiological gains (eg Clotfelter et al. 2007).

Overall, bicolor damselfish appear to respond to the approach of trumpetfish irrespective of their colouration. As a prey species, bicolor damselfish are under stronger selective pressure to survive encounters with potential predators (Dawkins and Krebs 1979), and have evolved the sensory and behavioral mechanisms to do so. A greater understanding of the factors that shape search-image formation in prey species, and how polymorphic predators can overcome these perceptual defences, therefore remain of interest for future study.

## Supplementary Material

araf059_suppl_Supplementary_Tables_S1-S2_Figures_S1-S3

## Data Availability

Analyses reported in this article can be reproduced using the data provided by Matchette et al. (2025).
