# Supplementary material for: Antagonistic effects of predator color morph abundance and saliency on prey anti-predator responses
Source: Behav Ecol. 2025 May 24;36(4):araf059. doi: 10.1093/beheco/araf059 (PMC12202312; doi:10.1093/beheco/araf059)
Supplement: araf059_suppl_Supplementary_Tables_S1-S2_Figures_S1-S3 [file araf059_suppl_supplementary_tables_s1-s2_figures_s1-s3.docx]

**Supplementary Materials**

*Trumpetfish Cadaver Image Transformation*

We analysed images of three fresh trumpetfish cadavers (one of each morph), each captured alongside a colour standard (ColorChecker Passport Photo 2; X-Rite, Grand Rapids, MI, USA) using a GoPro Hero 10 with default camera settings. These cadavers were obtained no longer than 20 min prior to photography, and we observed no noticeable colour changes in this time period. The right lateral side of each specimen was photographed from above at approximately 0.5 m, against a black fabric background and under diffuse sunlight.

First, the .jpg images were (i) linearised and (ii) the colour chart isolated and measured. We then used the greyscale measurements from the colour chart to (iii) standardise each image with respect to lighting conditions, by rescaling each colour channel to match the known greyscale values. The standardised images were then (iv) transformed into the colour space of the anemonefish *Amphiprion akindynos* (Cortesi et al., 2020; Hofmann et al., 2012; Siebeck & Marshall, 2007; Stieb et al., 2019). A cone-catch model implementing a polynomial transformation that maps from camera RGB to *A. akindynos* LMS (long, medium, and short wavelength cone quantal catches) colour space was generated using the ‘chart-based’ option in the MICA toolbox (Troscianko & Stevens, 2015) in ImageJ (v.1.53) (Schneider et al., 2012). The anemonefish spectral sensitivity curves used to create the cone-catch model were generated based on visual system data (Stieb et al., 2019) using the pavo package (Maia et al., 2013) in R (R Foundation for Statistical Computing, [www.R-project.org](http://www.R-project.org)). A mean RGB value was then (v) extracted from five regions (10 x 10 pixels) across the transformed trumpetfish image. These regions included two on the upper part of the trumpetfish’s snout, where their colouration is most prominent (*personal observation*), and three from their body (two close to the dorsal side, one closer to the ventral side), while ensuring that regions did not overlap with the black and white pigmented spots that are commonly present on trumpetfish. We created digital trumpetfish skins manually in GIMP (v.2.10.30), using the five primary colours extracted from each analysed trumpetfish images as a colour palette.

*Trumpetfish Cadaver Colour Analysis*

To check if, when printed, the digital skins matched the RGB values extracted from the photos of real trumpetfish, we printed a colour swatch of each of the five primary colours (for each morph). We applied two successive coats of non-toxic waterproof epoxy resin (mixed 1:1 with epoxy resin hardener) to each swatch, to ensure parity with the appearance of the final models, then photographed them alongside a colour standard under similar lighting conditions to the original photographs and using the same camera and set-up. Steps (i) to (v) from above were then repeated for the swatches, and the final RGB values compared to the “real” set by using the receptor-noise model to calculate achromatic (luminance; the dark–light component of the colour) and chromatic (pure colour, independent of luminance) just noticeable differences (JNDs) (Siddiqi et al., 2004; Vorobyev et al., 2001; Vorobyev & Osorio, 1998). One JND is defined as the minimum colour difference detectable by a given visual system. Chromatic JNDs were calculated from anemonefish LMS quantal catches using cone ratios of 2:2:1 (L:M:S) and weber fractions of 0.1 (for all cones); achromatic JNDs were calculated using the average of the M and L cones (the best approximation of luminance perception in this system) and a weber fraction of 0.08 (Siebeck et al., 2014; Stieb et al., 2019; van den Berg et al., 2020). If all comparisons did not generate JNDs that were less than 1, we adjusted the original RGB values (i.e., increased or decreased them), and the process was repeated. Once the colour palette for each skin was validated (in the methods outlined above), and the overall pattern created by eye using these colours matched our best approximation of trumpetfish, we printed the skins, wrapped them around the models and fixed them in place with super glue (Supplementary Figure S1).

*3D Model Colour Saliency Analysis*

One model of each morph was photographed alongside a colour standard (ColorChecker Passport Photo 2) from above at approximately 0.5 m, against a white fabric background and under diffuse sunlight. Each image was transformed to anemonefish colour space using the same methods described above. After the background was removed in each image, we used the *getHistList* function in the *colordistance* package (Weller & Westneat, 2019) to generate a colour histogram of each image (n = 27 bins, across three colour channels). By binning the colours in each image, we reduced and normalised the amount of colour data present, which allowed us to compare between images directly while maintaining the identity of the colours that best represent an image and their relative proportions (Weller & Westneat, 2019). This process was repeated for each colour morph. Given that, from the perspective of the damselfish, models were viewed against a midwater backdrop, we compared the colour histograms of each model image with a set of 50 “midwater” images, which were also transformed to anemonefish colour space and binned. These images, each a different hue of blue (Supplementary Figure S2), were chosen by eye and were deemed to reflect the natural diversity of midwater colour. The *getColorDistanceMatrix* function was then used to compare the colour distribution in each morph image with that in each of the blue images (n = 50 image comparisons per morph). Specifically, we implemented the “earth mover’s distance” method, which computes the ‘effort’ required to transform one distribution to another, accounting for both the amount in each colour bin as well as the relative difference between colour bins (Weller & Westneat, 2019).

*3D Model Reeling Speed*

Using the stereo camera videos, we extracted the position of each model at multiple increments across the presentation, to ensure that the speed of reeling did not differ between treatments. Two linear mixed models (LMMs) were used to compare the mean speed (log transformation) and the variation in speed (i.e., standard deviation; log transformation) for each morph treatment (nominal fixed effect). Colony ID was provided as a random effect. The mean (± 1 SD) model speeds of the treatments were: brown trumpetfish = 0.25 ± 0.05 m/s; blue trumpetfish = 0.26 ± 0.06 m/s; yellow trumpetfish = 0.25 ± 0.05 m/s. We found that neither the model speed (LRT = 0.60, df = 2, p = 0.740) nor the variation in model speed (LRT = 0.09, df = 2, p = 0.956) differed significantly between morph treatments.

**
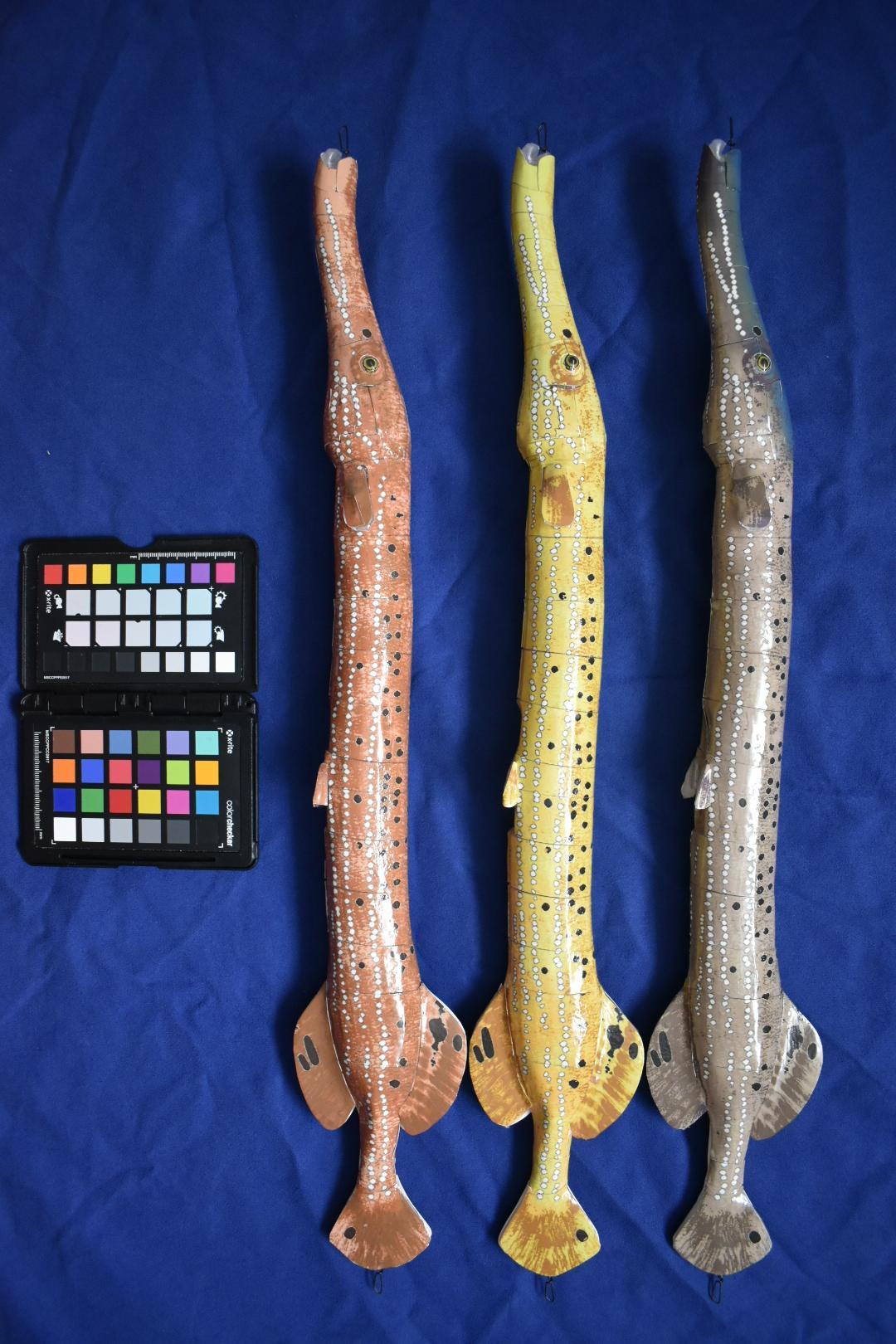
Figures**

**Figure S1.** An example of each of the three trumpetfish colour morph models used in the experiment: the blue morph (top), the yellow morph (middle) and the brown morph (bottom). To reduce pseudo-replication, we printed three models for each of the three colour morph treatments (nine models in total).

**
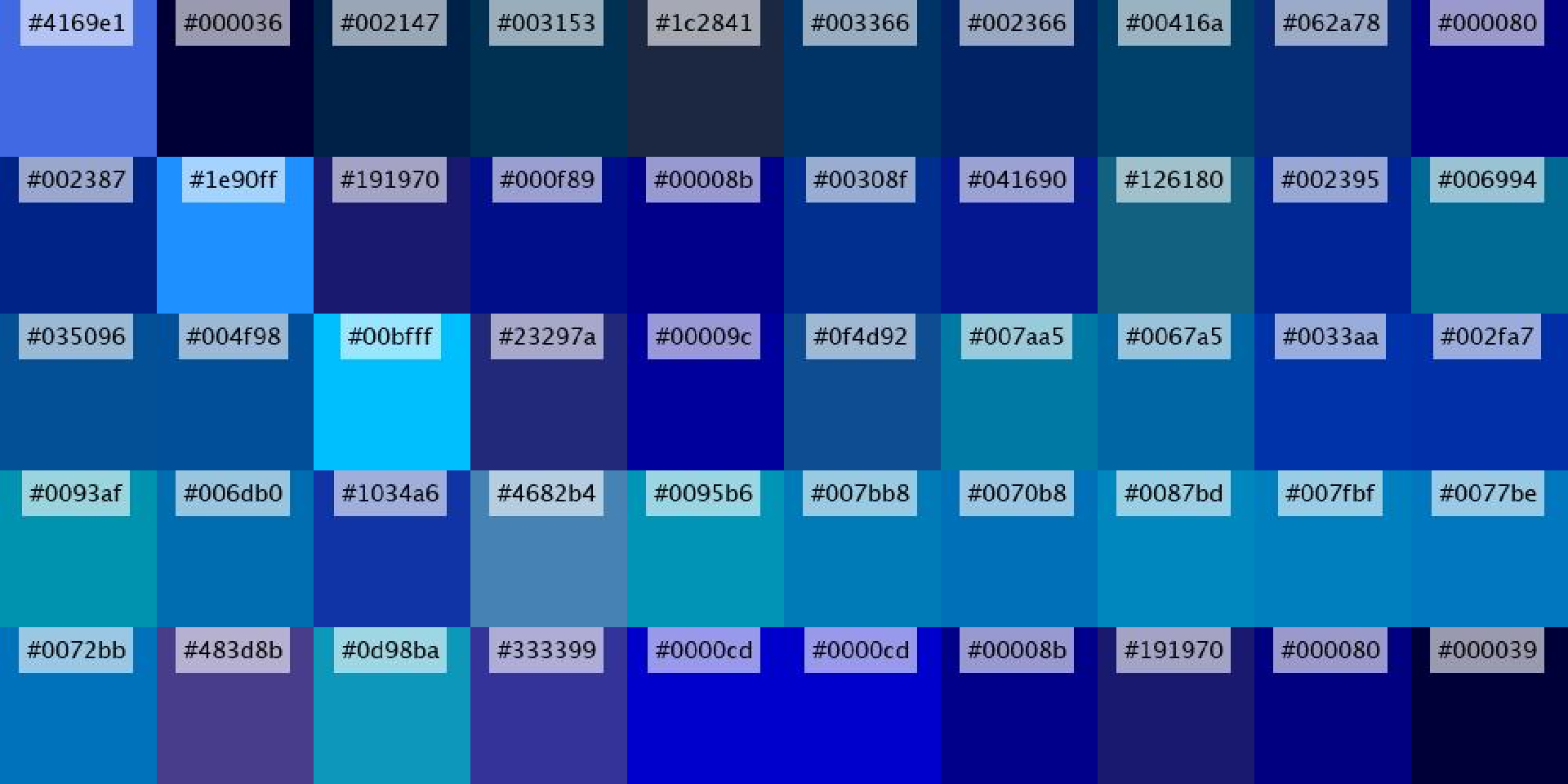
**

**Figure S2.** The palette of untransformed blue hues, and their corresponding hexcodes, which were compared to the colour distribution of each trumpetfish colour morph to assess their relative saliency. Like the trumpetfish model images, each blue was transformed into anemonefish colour space (our species proxy) before comparison.

**
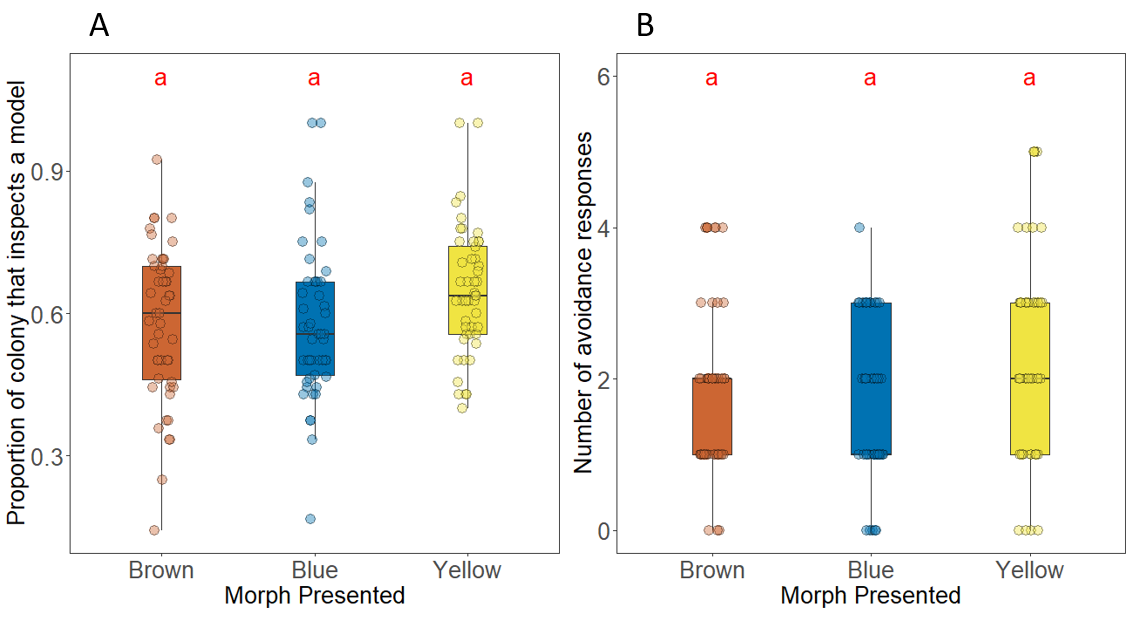
**

**Figure S3.** The proportion of damselfish within each colony (n = 40) that inspect each trumpetfish morph model (A) and the number of avoidance responses that each colony exhibits during a model presentation (B). Box plots show the median and 25th and 75th percentiles; the whiskers indicate the values within 1.5 times the interquartile range. The hollow circles represent the raw data points. Red letter labels to the right of each plot denote the pairwise comparisons between colour morphs, whereby morphs with the same letter do not statistically differ.

**Tables**

**Table S1**. List of locations visited to conduct transects (to quantify the abundance of each morph) and/or the behavioural experiment (to quantify behavioural responses of damselfish).

|  | **Location Name** | **Coordinates** | **Data Collected** |
| --- | --- | --- | --- |
| 1 | Playa Cas Abou | N 12.2283, W -69.0922 | Behavioural experiment; Morph abundance |
| 2 | Playa Porto Mari | N 12.2190, W -69.0863 | Behavioural experiment; Morph abundance |
| 3 | Playa Kokomo | N 12.1614, W -69.0046 | Behavioural experiment; Morph abundance |
| 4 | Play Boca Sami | N 12.1479, W -68.9988 | Morph abundance |
| 5 | Playa Wachi | N 12.1393, W -68.9980 | Morph abundance |
| 6 | Playa Piscadera | N 12.1223, W -68.9695 | Behavioural experiment; Morph abundance |
| 7 | Water Factory | N 12.1097, W -68.9542 | Morph abundance |
| 8 | Double Reef | N 12.1077, W -68.9499 | Morph abundance |
| 9 | Playa Marie Pompoen | N 12.0892, W -68.9045 | Morph abundance |


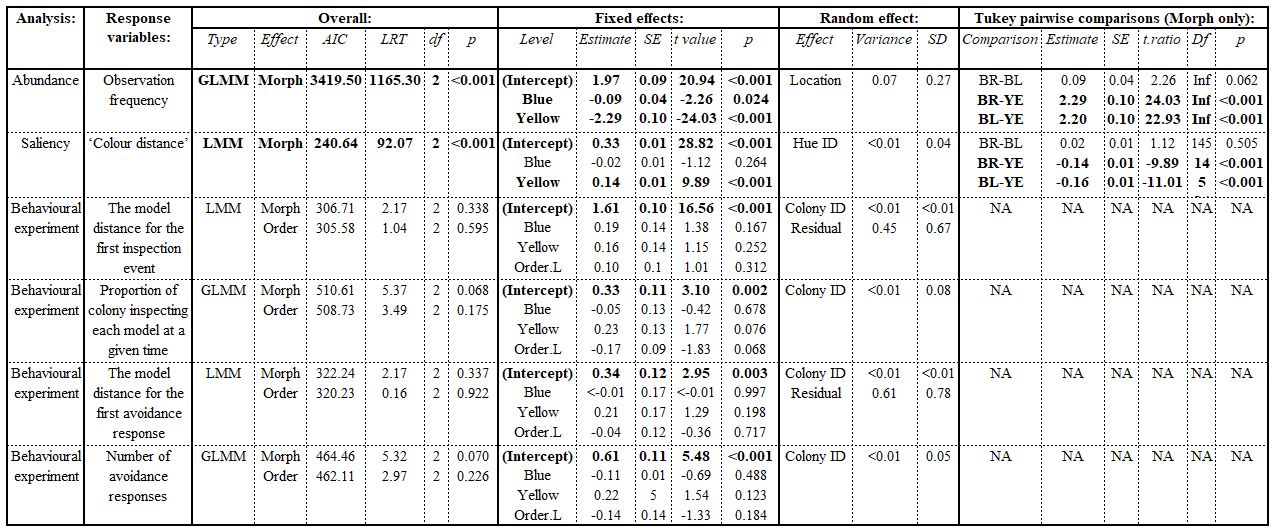
**Table S2**. The statistical output for linear mixed models (LMMs) and generalised linear mixed models (GLMMs) used throughout the investigation. Each response measure involved Morph (the trumpetfish colour morph) as a fixed effect, while the behavioural experiment also included Order (the order that models were presented) as a fixed effect. Location was included as a random effect for the abundance analysis, Hue ID was included as a random effect for the saliency analysis, and Colony ID was included as a random effect for the behavioural experiment analysis. Under fixed effects, the Intercept refers to the “Brown” trumpetfish colour morph, which was used as the comparative reference for the other fixed effects. Models that were significantly influenced by trumpetfish colour morph are highlighted in bold, as are significant pairwise comparisons.
